# Associations of Serum Uric Acid and *SLC2A9* Variant with Depressive and Anxiety Disorders: A Population-Based Study

**DOI:** 10.1371/journal.pone.0076336

**Published:** 2013-10-29

**Authors:** Tanica Lyngdoh, Murielle Bochud, Jennifer Glaus, Enrique Castelao, Gerard Waeber, Peter Vollenweider, Martin Preisig

**Affiliations:** 1 Institute of Social and Preventive Medicine (IUMSP), Lausanne University Hospital (CHUV), Lausanne, Switzerland; 2 Department of Psychiatry, CHUV, Lausanne, Switzerland; 3 Department of Medicine, Internal Medicine, CHUV and Faculty of Biology and Medicine, Lausanne, Switzerland; University of Illinois at Chicago, United States of America

## Abstract

**Background:**

Limited information exists regarding the association between serum uric acid (SUA) and psychiatric disorders. We explored the relationship between SUA and subtypes of major depressive disorder (MDD) and specific anxiety disorders. Additionally, we examined the association of *SLC2A9 rs6855911* variant with anxiety disorders.

**Methods:**

We conducted a cross-sectional analysis on 3,716 individuals aged 35–66 years previously selected for the population-based CoLaus survey and who agreed to undergo further psychiatric evaluation. SUA was measured using uricase-PAP method. The French translation of the semi-structured Diagnostic Interview for Genetic Studies was used to establish lifetime and current diagnoses of depression and anxiety disorders according to the DSM-IV criteria.

**Results:**

Men reported significantly higher levels of SUA compared to women (357±74 µmol/L vs. 263±64 µmol/L). The prevalence of lifetime and current MDD was 44% and 18% respectively while the corresponding estimates for any anxiety disorders were 18% and 10% respectively. A quadratic hockey-stick shaped curve explained the relationship between SUA and social phobia better than a linear trend. However, with regards to the other specific anxiety disorders and other subtypes of MDD, there was no consistent pattern of association. Further analyses using *SLC2A9 rs6855911* variant, known to be strongly associated with SUA, supported the quadratic relationship observed between SUA phenotype and social phobia.

**Conclusions:**

A quadratic relationship between SUA and social phobia was observed consistent with a protective effect of moderately elevated SUA on social phobia, which disappears at higher concentrations. Further studies are needed to confirm our observations.

## Introduction

Uric acid, a key end-product of purine metabolism, has a controversial role in human physiology acting potentially both as a pro-oxidant and selective antioxidant depending on its environmental milieu. It functions as an antioxidant primarily in extracellular compartment and as a pro-oxidant within the cells. While its role as a pro-oxidant in cardio-metabolic and cardiovascular diseases is well established, it has been observed that subjects with elevated serum uric acid (SUA) have lower prevalence of neurodegenerative diseases like multiple sclerosis, Parkinson's disease and Alzheimer's disease [1], [2], [3], [4]. Such a neuroprotective effect of SUA could be attributed to its antioxidant properties. Uric acid is capable of scavenging superoxide and hydroxyl radicals [5], [6] and accounts for over 60% of total antioxidant capacity in the human blood [5].

Though studies examining the role of SUA in affective disorders like major depressive disorder (MDD) and anxiety disorders are scant, the available evidence suggests a protective role of SUA in both depression [7], [8], [9] and anxiety [10]. The exact pathophysiologic mechanism linking SUA to these disease states is still unclear. Oxidative stress-related pathways have been implicated in depression and anxiety based mostly on experimental evidence [11], [12], [13], [14] and also several studies in humans [15], [16]. Hence, one possible mechanism by which SUA exerts its protective effect could be via a decrease in oxidative stress.

The few studies that have looked at the relation between SUA and MDD and anxiety disorders [7], [8], [9], [10] have methodological limitations which include small sample sizes and/or samples not representative of the general population. Hence, the current analysis explored the relationship between SUA and the lifetime and current occurrence of the commonest psychiatric disorders including the subtypes of MDD and specific anxiety disorders in individuals from a large population-based study. In addition, we examined the associations between a key variant of the *SLC2A9* gene which has been shown to have a strong association with SUA in genome-wide association studies [17], [18] and anxiety disorders.

## Methods

### Study population

Current analyses are performed within the PsyCoLaus study which is the psychiatric arm of the CoLaus study, a population-based study that assessed cardiovascular disease (CVD) risk factors and genetic variants associated with these conditions in Lausanne, Switzerland. Details of the CoLaus have been previously described [19]. Briefly, the random sampling procedure was based on a complete list of the Lausanne inhabitants aged 35–75 years in 2003, provided by the population register of the city. Sixty-seven percent of the CoLaus participants in the age range between 35 and 66 years (n = 5,535) accepted the psychiatric evaluation between 2004 and 2008, which resulted in a sample of 3,716 individuals who underwent both the medical and psychiatric exam [20]. Ninety-two percent of them were Caucasians. The gender distribution of the PsyCoLaus sample (47% males) did not differ significantly from that of the general population in the same age range. Although the youngest 5-year band of the cohort was underrepresented and the oldest 5-year band overrepresented, participants of PsyCoLaus (mean age 50.9±8.8 years) and individuals who refused to participate revealed comparable scores on the General Health Questionnaire (GHQ-12 [21]; French translation [22]), a self-rating instrument completed at the somatic exam. Both CoLaus and PsyCoLaus were approved by the Ethics Committee of the University of Lausanne. A written informed consent was obtained only from individuals without any apparent disability or handicap that might suggest the participant's incapability to participate in the study.

### Study procedure and measurements

#### Evaluations within the CoLaus study

Participants of the CoLaus study were seen in the outpatient clinic of the Centre Hospitalier Universitaire Vaudois (CHUV) in the morning after an overnight fast. They were asked to continue taking their medications as usual. The examination included a detailed health questionnaire, physical examination with anthropometric measures by trained and certified field workers and laboratory testing. Information on drugs that influence uric acid was assessed by recording all the prescribed drugs taken by the participants and was considered as present if participants were using any drugs including acetylsalicylic acid, diuretics, angiotensin converting enzymes inhibitors, angiotensin receptor blockers and other drugs known to induce hyperuricemia and hypouricemics (e.g., allopurinol, probenecid etc.). Blood pressure was measured in triplicate on the left arm and blood pressure values are the mean between the last two readings. Most clinical assays were performed by the CHUV Clinical Laboratory on fresh fasting blood samples. Glucose was measured by glucose dehydrogenase (2.1% - 1.0% maximum inter and intra-batch coefficients of variation); serum and urinary creatinine by Jaffe kinetic compensated method (2.9% - 0.7%) and uric acid by uricase-PAP (1.0% - 0.5%). Glomerular filtration rate (GFR) was estimated using the abbreviated Modification of the Diet in Renal Disease (MDRD) formula: 186×(serum creatinine [µmol/L]/88.4) (−1.154)×age (−0.203)×F, where F = 1 for men and F = 0.742 for women [23].

Hypertension was defined as mean systolic blood pressure of ≥140 mmHg or mean diastolic blood pressure of ≥90 mmHg and/or presence of anti-hypertensive medication. A diagnosis of diabetes was made if fasting plasma glucose was greater than or equal to 7.0 mmol/l or presence of oral hypoglycaemic or insulin treatment.

#### Evaluations within PsyColaus study

Diagnostic information was collected using the semi-structured Diagnostic Interview for Genetic Studies (DIGS) [24]. The DIGS was developed by the NIMH Molecular Genetics Initiative to obtain a more precise assessment of phenotypes through wide spectrum of DSM-IV Axis I criteria. The French translation of the DIGS [25] used in this study was jointly developed by the Department of Psychiatry of Lausanne and the INSERM in Paris and had several modifications. Additional questions were added to the depression section in order to elicit criteria for atypical depression features (leaden paralysis, long-standing patterns of interpersonal rejection sensitivity, mood reactivity). Similarly, a section on generalized anxiety disorder (GAD) was added using questions from the Schedule for Affective Disorders and Schizophrenia-Lifetime (SADS-LA) version and the brief phobia chapter of the DIGS was replaced by the corresponding more extensive chapters from the SADS-LA [26]. The French version of the DIGS had excellent inter-rater reliability for kappa and Yule's Y coefficient for major mood disorders [27] but 6-week test-retest reliability was slightly lower [27]. Similarly, the French translation of the SADS-LA revealed satisfactory test-retest reliability for anxiety disorders [28]. In our own reliability study we found excellent or perfect inter-rater reliability for all specific anxiety disorders, whereas the 6-week test-retest reliability estimates were in the fair or good range [29]. Lifetime and current (at the time of interview) diagnoses of depression and anxiety disorders were assigned according to DSM-IV criteria. The specifier for atypical depression features according to DSM-IV requires mood reactivity (the capacity to be cheered up when presented with positive events) and at least two of the following four symptoms: 1) increased appetite or significant weight gain, 2) hypersomnia, 3) leaden paralysis (i.e., heavy, leaden feelings in arms or legs), and 4) a long-standing pattern of interpersonal rejection sensitivity. According to Angst et al. [30], we used these criteria in a non-hierarchical way (depressive episode meeting at least 3 out of the 5 DSM-IV criteria of the atypical depression features specifier). For melancholic depression features, we applied the DSM-IV specifier which requires either a loss of energy or a lack of mood reactivity and 3 out of the following 5 symptoms: 1) depression regularly worse in the morning, 2) early morning awakening, 3) psychomotor retardation or agitation, 4) decreased appetite (we did not consider weight loss as a criterion) and 5) excessive guilt. We could not take into account the 6^th^ DSM-IV criterion “distinct quality of depressed mood” because it was not assessed in the DIGS. Following Angst et al. [30], MDD was subtyped according to the history of atypical or melancholic features into four subtypes 1) MDD with atypical features only, 2) MDD with melancholic features only, 3) combined MDD with atypical and melancholic features simultaneously or during distinct episodes and 4) unspecified MDD with neither atypical, nor melancholic features. All interviews were conducted by psychologists and psychiatrists trained over a two-month period. Their training included rating tapes and supervised co-ratings. Each interview and diagnostic assignment was reviewed by an experienced senior psychologist.

Socio-economic status was assessed using the Hollingshead scale [31]. Smoking was defined as current or past history of regular daily consumption of at least 10 cigarettes. Alcohol consumption was defined as glasses per typical week of consumption.

#### Genotyping

Nuclear DNA was extracted from whole blood for whole genome scan analysis. Genotyping was performed using Affymetrix 500K SNP chip, as recommended by the manufacturer (Affymetrix, Inc., Santa Clara, California, USA) on all participants of the CoLaus study. Persons with less than 95% genotyping efficiency overall (or <90% efficiency on either array; n = 399) and persons with possible gender inconsistencies (n = 5) were removed. Monomorphic single nucleotide polymorphisms (SNPs), SNPs with less than 70% genotyping efficiency, SNPs with minor allele frequency less than 1%, and/or not in the Hardy-Weinberg proportions were excluded. The SNP *rs6855911* in the *SLC2A9* gene was considered for the present analysis.

### Statistical analysis

All tests were performed using Stata 11 (StataCorp, College Station, TX, USA). Continuous variables were summarized as mean (standard deviation) or median (interquartile range) while categorical variables as number of subjects and percentages. We used t test (or Wilcoxon rank sum test) and χ2 test to compare the distribution of covariates according to sex. We described the distribution of MDD and anxiety disorders across sex-specific SUA quintiles and across genotypes of *rs6855911* within the *SLC2A9* gene and assessed for trends across these groups by using a non-parametric test which is an extension of the Wilcoxon rank-sum test [32]. These quintiles were generated separately in men and women, which leads to an equal proportion of men and women across quintiles.

We constructed multiple logistic regression models to examine the associations of SUA as independent variable of interest with MDD, MDD subtypes or specific anxiety disorders as dependent variable, one at a time. The predictor variable SUA was modeled as a continuous variable. Co-variates that were known to potentially influence the associations such as age, sex, socio-economic status, smoking, alcohol consumption, diabetes, hypertension, GFR and drugs that influence SUA were included in the models. Because depression and anxiety tend to co-exists [33], [34], we additionally adjusted for any anxiety disorder (in the association between SUA and depression) and MDD (in the association between SUA and anxiety). Furthermore, we included a quadratic term for SUA (i.e. SUA squared) in the models to account for non-linear relationship and used a likelihood ratio test (LRT) to compare model assuming a linear trend for SUA with another estimating quadratic fit; we maintained a quadratic model if the quadratic term was significant and P-value from the LRT was <0.05. Stratified analyses by sex were also conducted. We presented the quadratic relationship between SUA and social phobia graphically by plotting the fitted predicted probabilities after adjusting for the co-variates across SUA levels and by presenting the distribution of lifetime social phobia in men and women across genotypes of *SLC2A9 rs6855911* variant.

To test the potential modification of this association by sex, we included a multiplicative interaction parameter between sex and SUA into the logistic models. For models which included a quadratic term for SUA, interaction by sex was determined by LRT comparing a model with the multiplicative interaction parameters (SUA*sex and SUA squared*sex) to one without. We also performed logistic regression analyses where we further modeled *SLC2A9 rs6855911* as a score (where 0 = homozygote for non-risk allele, 1 = heterozygote and 2 = homozygote for risk allele) with anxiety disorders. The results from the genetic analyses serve to validate findings from logistic models using phenotypic SUA. We also tested the sex-by-genotype interaction for its effect on anxiety disorders in models including both men and women.

## Results

**Table 1** characterizes the demographic and clinical characteristics of the participants according to sex. SUA was significantly higher in men (357±74 µmol/L) than in women (263±64 µmol/L). Men had significantly higher prevalence of reported alcohol consumption and smoking compared to women. The overall prevalence estimates of diabetes and hypertension in the study population were 5% and 28% respectively, with significantly higher prevalence in men. The frequency of mental disorders in the overall population was high, with prevalence of lifetime and current MDD being 44% and 18% respectively. The corresponding prevalence estimates for any anxiety disorders were 18% and 10% respectively. Women consistently showed significantly higher lifetime as well as current prevalence of all subtypes of MDD and specific anxiety disorders (p<0.001 in almost all cases).

**Table 1:** Characteristics of the PsyCoLaus participants.

	Overall (N = 3716)		Men (N = 1748)		Women (N = 1968)		
	N	% or mean (s.d.)	N	% or mean (s.d.)	N	% or mean (s.d.)	*P*
**Age (years)** *	3716	50.9(8.8)	1748	50.5(8.8)	1968	51.3(8.8)	0.006
**Alcohol consumption** §	3716	3.0(0.0–8.0)	1748	6.0(2.0–14.0)	1968	2.0(0.0–5.0)	<0.001
**Daily smoking (>10 cigarettes)**	1655	44.5	869	49.7	786	39.9	<0.001
**Diabetes**	194	5.2	139	8.0	55	2.8	<0.001
**Hypertension**	1046	28.2	588	33.6	458	23.3	<0.001
**Drugs influencing uric acid levels**	471	13.8	247	15.3	224	12.4	0.014
**Serum uric acid (µmol/L)** *	3710	307.1(83.5)	1744	357.2(73.7)	1966	262.6(64.3)	<0.001
**GFR (ml/min/1.73 m^2^)** *	3709	84.4(15.8)	1743	87.2(0.4)	1966	81.9(0.3)	<0.001
***Lifetime pyschiatric disorders***							
*MDD*	*1625*	*43.7*	*565*	*32.3*	*1060*	*53.9*	*<0.001*
Combined MDD	218	5.9	61	3.5	157	8.0	<0.001
Atypical MDD	236	6.4	70	4.0	166	8.4	<0.001
Melancholic MDD	449	12.1	151	8.6	298	15.1	<0.001
Unspecified MDD	722	19.4	283	16.2	439	22.3	<0.001
*Any anxiety disorder*	*660*	*17.8*	*225*	*12.9*	*435*	*22.1*	*<0.001*
GAD	85	2.3	29	1.7	56	2.9	0.043
Panic disorder	118	3.2	30	1.7	88	4.5	<0.001
Agoraphobia	139	3.7	32	1.8	107	5.4	<0.001
Social phobia	444	12.0	162	9.3	282	14.3	<0.001
***Current psychiatric disorders***							
*MDD*	*660*	*17.8*	*236*	*13.5*	*424*	*21.5*	*<0.001*
Combined MDD	106	2.9	30	1.7	76	3.9	<0.001
Atypical MDD	98	2.6	31	1.8	67	3.4	0.002
Melancholic MDD	198	5.3	66	3.8	132	6.7	<0.001
Unspecified MDD	258	6.9	109	6.2	149	7.6	0.110
*Any anxiety disorder*	*370*	*10.0*	*118*	*6.8*	*252*	*12.8*	*<0.001*
GAD	0	0.0	0	0.0	0	0.0	NA
Panic disorder	1	0.0	0	0.0	1	0.1	NA
Agoraphobia	88	2.4	21	1.2	67	3.4	<0.001
Social phobia	294	7.9	98	5.6	196	10.0	<0.001

§ median (interquartile range);

* mean (standard deviation).

GFR = glomerular filtration rate; MDD = major depressive disorder; and GAD = generalized anxiety disorder.

**Table 2** describes the distribution of the lifetime and current prevalences of MDD and anxiety disorders across sex-specific quintiles of SUA in the overall sample. A significant negative linear trend was observed for anxiety disorder, in general, and for social phobia, in particular, suggesting lower prevalence of lifetime and current social phobia at higher levels of SUA. However, this decreasing trend in prevalence of social phobia was only up to a certain level of SUA after which an increase in prevalence was detected. Testing for comparison of fit of linear versus quadratic model indicated that a quadratic curve might explain the relationship better than a linear trend. In stratified analysis by sex (**Tables S1 and S2**), a hockey-stick shaped relationship (i.e. lowest prevalence in the fourth quartile) between SUA and lifetime and current social phobia was observed in women, although the quadratic test for trend was not statistically significant (P-value for quadratic trend = 0.153 and 0.087 respectively). A hockey-stick shaped relationship also tended to be present in men (P-value for quadratic trend = 0.095 and 0.210 respectively), although less clear than in women, possibly owing to the small number of cases of social phobia in each SUA quintile. **Figure S1** depicts the quadratic trend between SUA and social phobia based on adjusted predicted probabilities of social phobia across SUA levels from a multiple logistic regression model.

**Table 2:** Distribution of psychiatric disorders across sex-specific quintiles of SUA.

SUA (mean, µmol/L)	Q1 224	Q2 273	Q3 306	Q4 343	Q5 422	P-trend
	N(%)	N(%)	N(%)	N(%)	N(%)	(Test statistic)
**Lifetime pyschiatric disorders**						
*MDD*	*348(47.2)*	*323(45.4)*	*296(40.8)*	*259(40.1)*	*251(42.5)*	*0.012(−2.51)* *
Combined MDD	45(6.1)	44(6.2)	46(6.3)	33(5.1)	35(5.9)	0.624(−0.49)
Atypical MDD	46(6.2)	45(6.3)	39(5.4)	44(6.8)	40(6.8)	0.623(0.49)
Melancholic MDD	104(14.1)	94(13.2)	76(10.5)	64(9.9)	70(11.9)	0.038(−2.07)
Unspecified MDD	153(20.7)	140(19.7)	135(18.6)	118(18.3)	106(18.0)	0.144(−1.46)
*Any anxiety disorder*	*162(22.0)*	*126(17.9)*	*130(18.0)*	*102(15.9)*	*103(17.6)*	*0.015(−2.43)* *
GAD	15(2.0)	16(2.3)	14(1.9)	16(2.5)	17(2.9)	0.316(1.00)
Panic disorder	24(3.3)	17(2.4)	30(4.2)	26(4.0)	14(2.4)	0.965(0.04)
Agoraphobia	26(3.5)	26(3.7)	34(4.7)	29(4.5)	20(3.4)	0.735(0.34)
Social phobia	126(17.1)	84(11.9)	84(11.7)	55(8.6)	70(11.9)	<0.001(−3.62)*
**Current psychiatric disorders**						
*MDD*	163(22.1)	126(17.7)	130(17.9)	117(18.1)	121(20.5)	0.469(−0.72)
Combined MDD	24(3.3)	21(3.0)	23(3.2)	14(2.2)	24(4.1)	0.764(0.30)
Atypical MDD	20(2.7)	22(3.1)	16(2.2)	18(2.8)	21(3.6)	0.540(0.61)
Melancholic MDD	55(7.5)	43(6.0)	38(5.2)	34(5.3)	27(4.6)	0.020(−2.32)
Unspecified MDD	64(8.7)	40(5.6)	53(7.3)	51(7.9)	49(8.3)	0.700(0.38)
*Any anxiety disorder*	97(13.1)	71(10.0)	78(10.8)	50(7.7)	54(9.2)	0.005(−2.83)*
GAD	0(0.0)	0(0.0)	0(0.0)	0(0.0)	0(0.0)	NA
Panic disorder	0(0.0)	1(0.1)	0(0.0)	0(0.0)	0(0.0)	NA
Agoraphobia	16(2.2)	17(2.4)	23(3.2)	18(2.8)	10(1.7)	0.869(−0.17)
Social phobia	85(11.5)	55(7.7)	59(8.1)	34(5.3)	45(7.6)	0.001(−3.21)*

MDD =  major depressive disorder; GAD = generalized anxiety disorder; and SUA =  serum uric acid.

* P-value <0.05 for quadratic trend tested by a crude logistic model which included a quadratic term (SUA squared) for continuous value of SUA.

**Table 3** shows the associations of SUA with MDD and anxiety disorders in the overall sample either from models with or without the quadratic term for SUA depending on whether quadratic or linear model was a better fit. Both crude and adjusted analyses showed significant inverse associations of SUA with lifetime and current social phobia. With regards to the other specific anxiety disorders and subtypes of MDD, we found no consistent pattern of association. Although we did not observe any significant interaction between sex and SUA for any of the lifetime and current psychiatric disorders, separate analysis in males and females are presented in **Tables S3 and S4**.

**Table 3:** Crude and adjusted logistic regression analysis of SUA (per 100 µmol/L) and psychiatric disorders in overall sample.

	Crude OR(95% CI)				Adjusted OR(95% CI)				
Pyschiatric disorders	SUA	Pvalue_SUA_	SUA^2^	Pvalue_SUA2_	SUA	Pvalue_SUA_	SUA^2^	Pvalue_SUA2_	P_interaction_
**Lifetime pyschiatric disorders**									
*MDD*	*0.66(0.60–0.72)*	*<0.001*	*1.14(1.06–1.22)*	*<0.001*	*0.94(0.84–1.05)*	*0.274*	*-*	*-*	0.308
Combined MDD	0.71(0.59–0.86)	<0.001	-	-	1.01(0.80–1.27)	0.958	-	-	0.336
Atypical MDD	0.81(0.68–0.97)	0.019	-	-	1.11(0.88–1.38)	0.366	-	-	0.688
Melancholic MDD	0.72(0.63–0.82)	<0.001	-	-	0.86(0.72–1.02)	0.089	-	-	0.260
Unspecified MDD	0.85(0.77–0.95)	0.003	-	-	0.96(0.84–1.10)	0.577	-	-	0.537
*Any anxiety disorder*	*0.70(0.63–0.78)*	*<0.001*	*1.11(1.02–1.21)*	*0.019*	*0.89(0.77–1.03)*	*0.129*	*-*	*-*	0.983
GAD	0.93(0.71–1.22)	0.615	-	-	1.06(0.75–1.51)	0.737	-	-	0.532
Panic disorder	0.72(0.56–0.92)	0.008	-	-	1.06(0.78–1.46)	0.700	-	-	0.735
Agoraphobia	0.73(0.58–0.91)	0.005	-	-	0.97(0.73–1.28)	0.811	-	-	0.722
Social phobia	0.67(0.59–0.77)	<0.001	1.15(1.04–1.27)	0.008	0.79(0.66–0.93)	0.005	1.12(1.01–1.24)	0.029	0.873^§^
**Current psychiatric disorders**									
*MDD*	*0.78(0.70–0.87)*	*<0.001*	*1.11(1.02–1.21)*	*0.012*	*1.04(0.90–1.19)*	0.610	-	-	0.841
Combined MDD	0.79(0.62–1.01)	0.063	-	-	1.16(0.86–1.58)	0.334	-	-	0.670
Atypical MDD	0.90(0.70–1.15)	0.392	-	-	1.19(0.87–1.64)	0.271	-	-	0.814
Melancholic MDD	0.67(0.55–0.81)	<0.001	-	-	0.78(0.61–1.00)	0.054	-	-	0.556
Unspecified MDD	0.97(0.83–1.13)	0.710	-	-	1.11(0.91–1.37)	0.286	-	-	0.921
*Any anxiety disorder*	*0.66(0.57–0.75)*	*<0.001*	*1.14(1.02–1.27)*	*0.024*	*0.83(0.69–0.99)*	*0.045*	*-*	*-*	0.075
GAD*	NA	NA	NA	NA	NA	NA	NA	NA	NA
Panic disorder*	NA	NA	NA	NA	NA	NA	NA	NA	NA
Agoraphobia	0.65(0.49–0.87)	0.004	-	-	0.84(0.59–1.21)	0.352	-	-	0.948
Social phobia	0.65(0.56–0.75)	<0.001	1.22(1.09–1.37)	0.001	0.77(0.63–0.94)	0.009	1.19(1.06–1.34)	0.003	0.737^§^

MDD = major depressive disorder; GAD = generalized anxiety disorder; and SUA = serum uric acid.

If P value for the quadratic term (SUA^2^) is significant, ORs derived from models with quadratic term are presented.

Adjusted for age, sex, socio-economic status, alcohol consumption, smoking, diabetes, hypertension, GFR (calculated according to Modification in Diet in Renal Disease equation) and drugs that influence uric acid and additionally for anxiety (in the association between SUA and depression) and depression (in the association between SUA and anxiety).

P_interaction_ = P value for multiplicative interaction parameter between SUA and sex; Interaction for models with a quadratic term was assessed using a likelihood ratio test (LRT) comparing a model which included SUA*sex and SUA^2^* sex and a model without; and §indicates P value of the LRT.

* Logistic regression not possible due to zero prevalence; NA = not available.

The next set of analyses explored the association of uric acid with anxiety disorders using *SLC2A9 rs6588911* variant instead of phenotypic values for SUA. Logistic regression models (**Table 4**) revealed significant interactions between sex and SUA as explained by the *SLC2A9 rs6855911* variant regarding any anxiety disorder (P-value for the interaction = 0.026 and 0.014 for lifetime and current disorders, respectively) and social phobia (P-value for the interaction = 0.033 and 0.015 for lifetime and current disorder, respectively). Therefore, we present all analyses regarding the association of the SUA-related *rs6855911* variant with anxiety disorders separately in men and women. While a significant positive association of the allele associated with higher SUA levels (*rs6855911* A allele) with lifetime and current social phobia was observed in men, a negative association, though not significant, was noticed in women. Considering that women have lower SUA levels than men at each *rs6855911* genotype (see below), the observed effect modification by sex is consistent with the quadratic association of SUA with social phobia in non-genetic analyses.

**Table 4:** Crude and adjusted logistic regression analysis of *SLC2A9 rs6855911* and psychiatric disorders according to sex.

		Men		Women		
		OR(95% CI)	P-value	OR(95% CI)	P-value	P_interaction_
**Lifetime pyschiatric disorders**						
*Any anxiety disorder*	Unadjusted	1.38(1.05–1.80)	0.019	0.94(0.78–1.14)	0.525	
	Adjusted	1.40(1.07–1.84)	0.015	0.97(0.80–1.17)	0.728	0.026
GAD	Unadjusted	1.22(0.58–2.57)	0.601	0.94(0.58–1.51)	0.797	
	Adjusted	1.22(0.57–2.62)	0.602	0.94(0.58–1.52)	0.793	0.520
Panic disorder	Unadjusted	1.57(0.76–3.26)	0.225	0.80(0.55–1.15)	0.224	
	Adjusted	1.50(0.72–3.14)	0.277	0.82(0.56–1.19)	0.298	0.128
Agoraphobia	Unadjusted	1.50(0.77–2.92)	0.230	1.00(0.71–1.42)	0.979	
	Adjusted	1.61(0.82–3.18)	0.168	1.03(0.72–1.47)	0.865	0.287
Social phobia	Unadjusted	1.43(1.05–1.96)	0.025	0.93(0.75–1.17)	0.559	
	Adjusted	1.44(1.05–1.99)	0.023	0.96(0.76–1.21)	0.720	0.033
**Current psychiatric disorders**						
*Any anxiety disorder*	Unadjusted	1.42(1.00–2.03)	0.053	0.82(0.65–1.04)	0.097	
	Adjusted	1.42(0.99–2.03)	0.055	0.84(0.66–1.06)	0.140	0.014
Agoraphobia	Unadjusted	1.15(0.54–2.45)	0.713	0.84(0.54–1.29)	0.421	
	Adjusted	1.19(0.56–2.57)	0.649	0.85(0.55–1.32)	0.477	0.466
Social phobia	Unadjusted	1.51(1.02–2.25)	0.042	0.82(0.63–1.06)	0.124	
	Adjusted	1.50(1.00–2.23)	0.049	0.83(0.64–1.09)	0.184	0.015

GAD = generalized anxiety disorder.

ORs represent the effect of *SLC2A9 rs6855911* used as a continuous score of 0, 1 and 2 corresponding to GG, AG and AA genotype respectively with AA being the genotype associated with elevated levels of serum uric acid.

Adjusted for age, sex, socio-economic status, alcohol consumption, smoking, diabetes, hypertension, GFR (calculated according to Modification in Diet in Renal Disease equation) and drugs that influence uric acid and additionally for anxiety (in the association between SUA and depression) and depression (in the association between SUA and anxiety).

P_interaction_ = P value for sex-by-genotype interaction for its effect on psychiatric disorders.

We present the distribution of SUA and anxiety disorders across SUA-related *rs6855911* variant separately in men and women in **Table 5**. In women, a negative (although not significant) linear trend of social phobia was noted across genotypes associated with increasing levels of SUA similar to that observed in Table S2 using phenotypic SUA levels. On the other hand, a significant positive linear trend was seen in men, which was in contrast to that observed in women and also to the analysis that used SUA phenotype (in Table S1). Further, we graphically displayed in **Figure S2** the distribution of SUA and prevalence of social phobia across genotypes of the variant *SLC2A9 rs6855911* separately in men and women. Both men and women who carried the AA genotype had elevated levels of SUA as compared to carriers of the GG genotype. While in women a lower prevalence of social phobia was found in carriers of the AA genotype associated with higher SUA levels, the opposite trend was observed in men. Further, we observed a P-value of 0.014 for a LRT for a quadratic trend using the genetic variable coded as 0, 1, 2, 3, 4 and 5 where 0 = GG in females, 1 = AG in females, 2 = AA in females, 3 = GG in males, 4 = AG in males and 5 = AA in males (data not shown). This coding was chosen because it ranks these 6 independent groups of people according to increasing lifelong SUA levels. Taken together, these results are consistent with a quadratic relationship between *SLC2A9* genotypes (i.e. genetically determined SUA levels) and social phobia, which was similar to what we observed in Figure S1.

**Table 5:** Distribution of SUA and psychiatric outcomes across genotypes of *SLC2A9 rs6855911* in men and women.

	GG	AG	AA	P-trend
**MEN (N)**	107	544	706	
**SUA (mean, SD) µmol/L**	317.96(76.11)	352.57(69.60)	367.63(74.75)	<0.001
**Lifetime pyschiatric disorders (N, %)**				
*Any anxiety disorders*	8(7.55)	62(11.44)	104(14.81)	0.014
GAD	1(0.94)	7(1.29)	11(1.57)	0.556
Panic disorder	0(0.00)	9(1.66)	14(1.99)	0.203
Agoraphobia	2(1.89)	7(1.29)	18(2.57)	0.211
Social phobia	6(5.66)	42(7.75)	77(10.98)	0.019
**Current psychiatric disorders (N, %)**				
*Any anxiety disorders*	5(4.67)	31(5.70)	59(8.36)	0.044
Agoraphobia	2(1.87)	5(0.92)	11(1.56)	0.694
Social phobia	3(2.80)	26(4.78)	49(6.94)	0.034
**WOMEN (N)**	108	653	780	
**SUA (mean, SD) µmol/L**	220.73(50.36)	251.60(61.36)	278.87(62.69)	<0.001
**Lifetime pyschiatric disorders (N, %)**				
*Any anxiety disorders*	23(21.50)	161(24.73)	170(21.88)	0.463
GAD	3(2.80)	20(3.07)	21(2.70)	0.767
Panic disorder	5(4.67)	37(5.68)	30(3.87)	0.215
Agoraphobia	6(5.61)	37(5.68)	44(5.66)	0.996
Social phobia	18(16.82)	99(15.21)	112(14.41)	0.492
**Current psychiatric disorders (N, %)**				
*Any anxiety disorders*	19(17.59)	89(13.63)	92(11.79)	0.083
Agoraphobia	5(4.63)	23(3.52)	24(3.08)	0.404
Social phobia	16(14.81)	69(10.57)	73(9.36)	0.108

GAD = generalized anxiety disorder; and SUA = serum uric acid.

The results are expressed as numbers and percentages except for SUA which is expressed as mean and standard deviation.

Sex-by-genotype interaction were significant for its effect on lifetime and current any anxiety disorders and social phobia (Table 4).

## Discussion

To the best of our knowledge, this is by far the largest population-based study to assess the relationship of SUA with depressive and anxiety disorders. We found a significant quadratic (hockey-stick shaped) association between SUA with both lifetime and current social phobia suggesting that elevated levels of SUA are associated with lower frequency of social phobia only up to a certain concentration after which increasing SUA levels are no longer protective. It is interesting to note that SUA does not appear to be protective when in the range of hyperuricemia.

We observed a similar quadratic relationship between *SLC2A9 rs6855911* (associated with increasing levels of uric acid) and social phobia when keeping both men and women in the analysis. Similar to what was observed for the non-genetic association, the overall quadratic association of *rs6855911* genotypes with social phobia disappeared when stratifying the analyses by sex. Findings from genetic associations are generally considered to be free from the inherent problems of reverse causation and confounding. Recent genome wide association studies have identified *SLC2A9* gene, a putative hexose transporter, to be strongly associated with SUA [18], [35], [36]. *SLC2A9* gene explains a substantial proportion (1–6%) of the variance in SUA concentration [37]. Considering that genes are inherited at the time of conception from parents through random transmission, genetic associations of single nucleotide polymorphisms (SNPs) within or around the genes will be less likely to be influenced by problems of reverse causality or measurement errors inherent to conventional observational studies and more likely to produce robust causal inferences. The results of the genetic associations for social phobia using the *SLC2A9 rs6855911* variant serve to validate the findings of the observational analysis. It confirmed the finding that SUA does not confer protective effects beyond a certain concentration as evidenced from the negative association (i.e., those carrying the allele related to elevated levels of SUA were associated with decreased risk of social phobia) observed in women and the opposite association in men. Women have much lower SUA levels than men [38], due to the uricosuric effects of estrogen [39]. Our findings provide new insights which need to be replicated in other settings.

Very few studies have explored the association between SUA and anxiety disorders. The earliest study dating back to 1979 and carried out on 20 students aged between 18–30 years found levels of SUA to be significantly decreased during periods of anxiety [10]. Similar decreases in SUA levels in stressful situations have been observed. Trevisan et al. observed exposure to a natural disaster (major earthquake) in 578 healthy factory workers in Naples to be associated in the short term with a reduction and in the long-term with an increase in SUA levels [40]. More recently, a study in Japan found no association between SUA and post-traumatic stress disorder in 34 victims of the Tokyo Sarin attack. However, the authors do acknowledge that lack of association could have been due to the limited sample size and 5-year time gap between the event and the study [41]. Our finding of an inverse association between SUA level and social phobia (the most common anxiety disorders [42]), which is an indicator of permanent social stress, are consistent with previous studies that reported decreased SUA levels in periods of stress and anxiety. Similar findings were also documented from longitudinal studies on US navy men undergoing training as a part of the Navy Underwater Demolition Team [43], [44], [45]. SUA varied significantly with elevated levels during periods of rigorous training for challenging tasks that were eagerly awaited and decreased levels during periods of unanticipated intense psychological and physical stress. Moreover, a similar experiment by the same group on three medical investigators over six-month periods also revealed increases in SUA levels above baseline in situations prior to a physical challenge in which the associated stress is eagerly anticipated as a pleasant challenge [46].

Although three previous studies documented negative associations between SUA level and depression, we did not find such an association of SUA with MDD or its subtypes. In the first study of 826 individuals with age ranging from 16–86 years Wen et al found depressive patients (based on scores from the Hamilton Depression Scale (HAMD24)) to have significantly lower SUA levels as compared to patients with other mental disorders and healthy controls [9]. Similarly, a significant negative correlation between SUA and Zung Self-Rating Depression Scale scores was observed in another population-based (the Massa Lombarda Project) study on 106 subjects with a mean age of about 80 years [7]. The third study, a randomized controlled trial, among age and sex-matched 40 MDD based on the Hamilton Rating Scale for Depression scores and 36 healthy subjects before and after anti-depressive drug treatment found significantly lower SUA levels in newly diagnosed MDD subjects compared to healthy subjects with a reverse trend after 12 weeks of treatment [8]. Differences in the sampling or the use of different instruments to assess depression are likely to account for the different results in the present study as compared to the three previous studies. Indeed, two of the three previous studies included treated depressive subjects, who were likely to be more severely affected than the depressive sample of the present population-based study.

The exact mechanisms of action linking SUA with anxiety disorders still remains elusive. Oxidative stress has recently gained attention as one of the suggested mechanisms in the pathophysiology of MDD and anxiety disorders. Evidence from experimental studies clearly supports this hypothesis [11], [12], [13], [14]. These findings further extend to studies in humans where elevated oxidative stress levels have been observed in patients with depression [16], [47], [48], [49] and anxiety [15]. Oxidative stress characterized by an excess generation of reactive oxygen species can affect a number of physiological functions including the central nervous system. The brain has modest antioxidant levels and is particularly vulnerable to the detrimental effects of oxidative stress as the neuronal membrane containing a high proportion of polyunsaturated fatty acids is a key site for oxidative stress [50]. Uric acid, by virtue of its powerful anti-oxidant properties [5], may provide a defense against the oxidative stress [51]. In addition, recent observations have also attributed the protective effect of SUA to its ability to decrease blood-brain barrier permeability as well as a more direct effect on astroglial cells [52], [53]. However, the relation between SUA and anxiety may not be as straightforward as it appears, which is also reflected in the non-linear relationship observed in the current study. This is not surprising and probably relates to the complex and paradoxical role of uric acid in the “antioxidant – prooxidant urate redox shuttle” which states that “in a prooxidative environmental milieu the original antioxidant properties of uric acid paradoxically become prooxidant” [54]. Furthermore, it is difficult to distinguish whether lowered SUA levels observed in depressed and anxious subjects is a cause or consequence of the disease. As Wen and colleagues pointed out, it is unclear if depressed patients with low SUA levels are more likely to be challenged with free radical toxicity and inflammation or rather low SUA is due to the consumption of uric acid by radical interaction and damaged tissue encountered in anxiety or depression [9].

The strengths of this study are its population-based design, the large sample size, the face-to-face structured interview-based psychiatric diagnoses, the availability of detailed information on a number of potential confounders known to influence the association and the accessibility to genetic markers that allowed for validating the findings from phenotypic analyses. The potential limitations are: first, the cross-sectional nature of the study with the establishment of lifetime diagnoses of MDD and anxiety disorders which did not allow us to infer causality of non-genetic findings. However, the findings from the genetic analysis using *SLC2A9 rs6855911* corroborated the results from the phenotypic analysis (where SUA was used). Indeed, inferences drawn from genetic analyses are usually more robust and can be considered as causal. Second, there was a gap of about one year [median (interquartile range) being 1.2(1.1–1.3) years] between the evaluation of SUA and assessments of psychiatric phenotypes. However, evidence have shown that SUA taken 5 years apart have a high correlation (r = 0.50) [55] which makes it unlikely that the subjects would have very different levels of SUA after an interval of one year.

In conclusion, in this population-based sample, we found a quadratic (hockey-stick shaped) relation between SUA and social phobia, with both non-genetic and genetic associations, consistent with a potential protective effect of moderately elevated SUA on social phobia, which disappears when SUA levels reach the hyperuricemic zone. Since the use of genetic variants reflecting lifelong differences in SUA levels is usually robust to confounding and reverse causality, our results suggest that the observed association may be causal. However, prospective observational and experimental studies are needed to confirm our observations.

## Supporting Information

Figure S1 **A quadratic curve association between serum uric acid (SUA) and social phobia.** The dots and bars represent the adjusted probabilities along with 95%CI of social phobia across SUA levels. Solid line represents the fitted quadratic curve of the effect of SUA on social phobia. Adjusted for age, sex, socio-economic status, alcohol consumption, smoking, diabetes, hypertension, GFR (calculated according to Modification in Diet in Renal Disease equation), drugs that influence uric acid and depression.
(TIF)

Figure S2 **Distribution of serum uric acid (SUA) and current social phobia across genotypes of** ***SLC2A9 rs6855911*** **in men and women.** ♀ = women; ♂ = men. Red and blue barplots indicate mean SUA across the genotypes of SLC2A9 rs6855911 in women and men respectively. Black diamonds with bars indicate adjusted prevalence and 95% CI of social phobia across the genotypes GG, AG and AA of the SLC2A9 rs6855911 variant. Prevalence adjusted for age, sex, socio-economic status, alcohol consumption, smoking, diabetes, hypertension, GFR (calculated according to Modification in Diet in Renal Disease equation), drugs that influence uric acid and depression.
(TIF)

Table S1 **Distribution of psychiatric disorders across sex-specific quintiles of SUA in males.** MDD = major depressive disorder; GAD = generalized anxiety disorder; and SUA = serum uric acid. *P-value<0.05 for quadratic trend tested by a crude logistic model which included a quadratic term (SUA squared) for continuous value of SUA.
(DOCX)

Table S2 **Distribution of psychiatric disorders across sex-specific quintiles of SUA in females.** MDD = major depressive disorder; GAD = generalized anxiety disorder; and SUA = serum uric acid. *P-value<0.05 for quadratic trend tested by a crude logistic model which included a quadratic term (SUA squared) for continuous value of SUA.
(DOCX)

Table S3 **Crude and adjusted logistic regression analysis of SUA (per 100 µmol/L) and psychiatric disorders in males.** MDD = major depressive disorder; GAD = generalized anxiety disorder; and SUA = serum uric acid.If P value for the quadratic term (SUA2) is significant, ORs derived from models with quadratic term are presented.Adjusted for age, sex, socio-economic status, alcohol consumption, smoking, diabetes, hypertension, GFR (calculated according to Modification in Diet in Renal Disease equation) and drugs that influence uric acid and additionally for anxiety (in the association between SUA and depression) and depression (in the association between SUA and anxiety). *Logistic regression not possible due to zero prevalence; NA = not available.
(DOCX)

Table S4 **Crude and adjusted logistic regression analysis of SUA (per 100 µmol/L) and psychiatric disorders in females.** MDD = major depressive disorder; GAD = generalized anxiety disorder; and SUA = serum uric acid. If P value for the quadratic term (SUA2) is significant, ORs derived from models with quadratic term are presented. Adjusted for age, sex, socio-economic status, alcohol consumption, smoking, diabetes, hypertension, GFR (calculated according to Modification in Diet in Renal Disease equation) and drugs that influence uric acid and additionally for anxiety (in the association between SUA and depression) and depression (in the association between SUA and anxiety). *Logistic regression not possible due to zero prevalence; NA = not available.
(DOCX)

## References

[1] Church WH, Ward VL (1994) Uric acid is reduced in the substantia nigra in Parkinson's disease: effect on dopamine oxidation. Brain Res Bull 33: 419–425. 8124580 10.1016/0361-9230(94)90285-2

[2] de Lau LM, Koudstaal PJ, Hofman A, Breteler MM (2005) Serum uric acid levels and the risk of Parkinson disease. Ann Neurol 58: 797–800. 16240356 10.1002/ana.20663

[3] Kim TS, Pae CU, Yoon SJ, Jang WY, Lee NJ, et al (2006) Decreased plasma antioxidants in patients with Alzheimer's disease. Int J Geriatr Psychiatry 21: 344–348. 16534775 10.1002/gps.1469

[4] Toncev G, Milicic B, Toncev S, Samardzic G (2002) Serum uric acid levels in multiple sclerosis patients correlate with activity of disease and blood-brain barrier dysfunction. Eur J Neurol 9: 221–226. 11985629 10.1046/j.1468-1331.2002.00384.x

[5] Ames BN, Cathcart R, Schwiers E, Hochstein P (1981) Uric acid provides an antioxidant defense in humans against oxidant- and radical-caused aging and cancer: a hypothesis. Proc Natl Acad Sci U S A 78: 6858–6862. 6947260 10.1073/pnas.78.11.6858 PMC349151

[6] Davies KJ, Sevanian A, Muakkassah-Kelly SF, Hochstein P (1986) Uric acid-iron ion complexes. A new aspect of the antioxidant functions of uric acid. Biochem J 235: 747–754. 3753442 10.1042/bj2350747 PMC1146751

[7] Bove M, Carnevali L, Cicero AF, Grandi E, Gaddoni M, et al (2010) Psychosocial factors and metabolic parameters: is there any association in elderly people? The Massa Lombarda Project. Aging Ment Health 14: 801–806. 20635238 10.1080/13607861003713299 PMC2928408

[8] Chaudhari K, Khanzode S, Khanzode S, Dakhale G, Saoji A, et al (2010) Clinical correlation of alteration of endogenous antioxidant-uric acid level in major depressive disorder. Indian Journal of Clinical Biochemistry 25: 77–81. 23105889 10.1007/s12291-010-0016-z PMC3453017

[9] Wen S, Cheng M, Wang H, Yue J, Li G, et al (2012) Serum uric acid levels and the clinical characteristics of depression. Clin Biochem 45: 49–53. 22040815 10.1016/j.clinbiochem.2011.10.010

[10] Francis KT (1979) Psychologic correlates of serum indicators of stress in man: a longitudinal study. Psychosom Med 41: 617–628. 232553 10.1097/00006842-197912000-00003

[11] Bouayed J, Rammal H, Soulimani R (2009) Oxidative stress and anxiety: relationship and cellular pathways. Oxid Med Cell Longev 2: 63–67. 20357926 10.4161/oxim.2.2.7944 PMC2763246

[12] Hovatta I, Juhila J, Donner J (2010) Oxidative stress in anxiety and comorbid disorders. Neurosci Res 68: 261–275. 20804792 10.1016/j.neures.2010.08.007

[13] Hovatta I, Tennant RS, Helton R, Marr RA, Singer O, et al (2005) Glyoxalase 1 and glutathione reductase 1 regulate anxiety in mice. Nature 438: 662–666. 16244648 10.1038/nature04250

[14] Salim S, Asghar M, Taneja M, Hovatta I, Chugh G, et al (2011) Potential contribution of oxidative stress and inflammation to anxiety and hypertension. Brain Res 1404: 63–71. 21704983 10.1016/j.brainres.2011.06.024 PMC3402065

[15] Matsushita M, Kumano-Go T, Suganuma N, Adachi H, Yamamura S, et al (2010) Anxiety, neuroticism and oxidative stress: cross-sectional study in non-smoking college students. Psychiatry Clin Neurosci 64: 435–441. 20653910 10.1111/j.1440-1819.2010.02109.x

[16] Yanik M, Erel O, Kati M (2004) The relationship between potency of oxidative stress and severity of depression. Acta Neuropsychiatrica 16: 200–203. 26984307 10.1111/j.0924-2708.2004.00090.x

[17] Brandstatter A, Kiechl S, Kollerits B, Hunt SC, Heid IM, et al (2008) Sex-specific association of the putative fructose transporter SLC2A9 variants with uric acid levels is modified by BMI. Diabetes Care 31: 1662–1667. 18487473 10.2337/dc08-0349 PMC2494626

[18] Vitart V, Rudan I, Hayward C, Gray NK, Floyd J, et al (2008) SLC2A9 is a newly identified urate transporter influencing serum urate concentration, urate excretion and gout. Nat Genet 40: 437–442. 18327257 10.1038/ng.106

[19] Firmann M, Mayor V, Vidal PM, Bochud M, Pecoud A, et al (2008) The CoLaus study: a population-based study to investigate the epidemiology and genetic determinants of cardiovascular risk factors and metabolic syndrome. BMC Cardiovasc Disord 8: 6. 18366642 10.1186/1471-2261-8-6 PMC2311269

[20] Preisig M, Waeber G, Vollenweider P, Bovet P, Rothen S, et al (2009) The PsyCoLaus study: methodology and characteristics of the sample of a population-based survey on psychiatric disorders and their association with genetic and cardiovascular risk factors. BMC Psychiatry 9: 9. 19292899 10.1186/1471-244X-9-9 PMC2667506

[21] Goldberg DP (1972) The detection of psychiatric illness by questionnaire. Oxford: Oxford University Press.

[22] Bettschart W, Bolognini M (1996) Questionnaire de santé GHQ-12. In: Guelfi J, editor. L'évaluation clinique standardisée en psychiatrie Tome I. Boulogne: Médicales Pierre Fabre.

[23] Levey AS, Stevens LA, Schmid CH, Zhang YL, Castro AF 3rd, et al (2009) A new equation to estimate glomerular filtration rate. Ann Intern Med 150: 604–612. 19414839 10.7326/0003-4819-150-9-200905050-00006 PMC2763564

[24] Nurnberger JI Jr, Blehar MC, Kaufmann CA, York-Cooler C, Simpson SG, et al (1994) Diagnostic interview for genetic studies. Rationale, unique features, and training. NIMH Genetics Initiative. Arch Gen Psychiatry 51: 849–859; discussion 863–844. 7944874 10.1001/archpsyc.1994.03950110009002

[25] Leboyer M, Barbe B, Gorwood P, Teherani M, Allilaire JF, et al.. (1995) Interview Diagnostique pour les Etudes Génétiques Paris:INSERM.

[26] Endicott J, Spitzer RL (1978) A diagnostic interview: the schedule for affective disorders and schizophrenia. Arch Gen Psychiatry 35: 837–844. 678037 10.1001/archpsyc.1978.01770310043002

[27] Preisig M, Fenton BT, Matthey ML, Berney A, Ferrero F (1999) Diagnostic interview for genetic studies (DIGS): inter-rater and test-retest reliability of the French version. Eur Arch Psychiatry Clin Neurosci 249: 174–179. 10449592 10.1007/s004060050084

[28] Leboyer M, Maier W, Teherani M, Lichtermann D, D'Amato T, et al (1991) The reliability of the SADS-LA in a family study setting. Eur Arch Psychiatry Clin Neurosci 241: 165–169. 1790162 10.1007/BF02219716

[29] Rougemont-Buecking A, Rothen S, Jeanpretre N, Lustenberger Y, Vandeleur CL, et al (2008) Inter-informant agreement on diagnoses and prevalence estimates of anxiety disorders: direct interview versus family history method. Psychiatry Res 157: 211–223. 17881063 10.1016/j.psychres.2006.04.022

[30] Angst J, Gamma A, Benazzi F, Silverstein B, Ajdacic-Gross V, et al (2006) Atypical depressive syndromes in varying definitions. Eur Arch Psychiatry Clin Neurosci 256: 44–54. 16041559 10.1007/s00406-005-0600-z

[31] Hollingshead AB (1975) Four factor index of social status. New Haven, Department of Sociology, Yale University.

[32] Cuzick J (1985) A Wilcoxon-type test for trend. Stat Med 4: 87–90. 3992076 10.1002/sim.4780040112

[33] Hettema JM (2008) What is the genetic relationship between anxiety and depression? Am J Med Genet C Semin Med Genet 148C: 140–146. 18412101 10.1002/ajmg.c.30171

[34] Kessler RC, Nelson CB, McGonagle KA, Liu J, Swartz M, et al (1996) Comorbidity of DSM-III-R major depressive disorder in the general population: results from the US National Comorbidity Survey. Br J Psychiatry Suppl 17–30. 8864145

[35] Doring A, Gieger C, Mehta D, Gohlke H, Prokisch H, et al (2008) SLC2A9 influences uric acid concentrations with pronounced sex-specific effects. Nat Genet 40: 430–436. 18327256 10.1038/ng.107

[36] Li S, Sanna S, Maschio A, Busonero F, Usala G, et al (2007) The GLUT9 gene is associated with serum uric acid levels in Sardinia and Chianti cohorts. PLoS Genet 3: e194. 17997608 10.1371/journal.pgen.0030194 PMC2065883

[37] Le MT, Shafiu M, Mu W, Johnson RJ (2008) SLC2A9–a fructose transporter identified as a novel uric acid transporter. Nephrol Dial Transplant 23: 2746–2749. 18606621 10.1093/ndt/gfn349 PMC2734172

[38] Culleton BF, Larson MG, Kannel WB, Levy D (1999) Serum uric acid and risk for cardiovascular disease and death: the Framingham Heart Study. Ann Intern Med 131: 7–13. 10391820 10.7326/0003-4819-131-1-199907060-00003

[39] Nicholls A, Snaith ML, Scott JT (1973) Effect of oestrogen therapy on plasma and urinary levels of uric acid. Br Med J 1: 449–451. 4689833 10.1136/bmj.1.5851.449 PMC1588487

[40] Trevisan M, O'Leary E, Farinaro E, Jossa F, Galasso R, et al (1997) Short- and long-term association between uric acid and a natural disaster. Psychosom Med 59: 109–113. 9088046 10.1097/00006842-199703000-00001

[41] Tochigi M, Umekage T, Otani T, Kato T, Iwanami A, et al (2002) Serum cholesterol, uric acid and cholinesterase in victims of the Tokyo subway sarin poisoning: a relation with post-traumatic stress disorder. Neurosci Res 44: 267–272. 12413655 10.1016/s0168-0102(02)00146-3

[42] Kessler RC, McGonagle KA, Zhao S, Nelson CB, Hughes M, et al (1994) Lifetime and 12-month prevalence of DSM-III-R psychiatric disorders in the United States. Results from the National Comorbidity Survey. Arch Gen Psychiatry 51: 8–19. 8279933 10.1001/archpsyc.1994.03950010008002

[43] Rahe RH, Arthur RJ (1967) Stressful underwater demolition training. Serum urate and cholesterol variability. JAMA 202: 1052–1054. 6072605

[44] Rahe RH, Rubin RT, Arthur RJ, Clark BR (1968) Serum uric acid and cholesterol variability. A comprehensive view of underwater demolition team training. JAMA 206: 2875–2880. 5755010

[45] Zir LM, McHugh WB, Rahe RH, Arthur RJ, Rubin RT (1973) Renal excretion of uric acid. Alterations during stressful underwater demolition-team training. Arch Intern Med 132: 808–812. 4757252

[46] Rahe RH, Rubin RT, Arthur RJ (1974) The three investigators study. Serum uric acid, cholesterol, and cortisol variability during stresses of everyday life. Psychosom Med 36: 258–268. 4829620 10.1097/00006842-197405000-00009

[47] Cumurcu BE, Ozyurt H, Etikan I, Demir S, Karlidag R (2009) Total antioxidant capacity and total oxidant status in patients with major depression: impact of antidepressant treatment. Psychiatry Clin Neurosci 63: 639–645. 19674383 10.1111/j.1440-1819.2009.02004.x

[48] Irie M, Asami S, Ikeda M, Kasai H (2003) Depressive state relates to female oxidative DNA damage via neutrophil activation. Biochem Biophys Res Commun 311: 1014–1018. 14623283 10.1016/j.bbrc.2003.10.105

[49] Sarandol A, Sarandol E, Eker SS, Erdinc S, Vatansever E, et al (2007) Major depressive disorder is accompanied with oxidative stress: short-term antidepressant treatment does not alter oxidative-antioxidative systems. Hum Psychopharmacol 22: 67–73. 17299810 10.1002/hup.829

[50] Fendri C, Mechri A, Khiari G, Othman A, Kerkeni A, et al (2006) [Oxidative stress involvement in schizophrenia pathophysiology: a review]. Encephale 32: 244–252. 16910626 10.1016/s0013-7006(06)76151-6

[51] Klandorf H, Rathore DS, Iqbal M, Shi X, Van Dyke K (2001) Accelerated tissue aging and increased oxidative stress in broiler chickens fed allopurinol. Comp Biochem Physiol C Toxicol Pharmacol 129: 93–104. 11423382 10.1016/s1532-0456(01)00186-7

[52] Du Y, Chen CP, Tseng CY, Eisenberg Y, Firestein BL (2007) Astroglia-mediated effects of uric acid to protect spinal cord neurons from glutamate toxicity. Glia 55: 463–472. 17203476 10.1002/glia.20472

[53] Spitsin SV, Scott GS, Mikheeva T, Zborek A, Kean RB, et al (2002) Comparison of uric acid and ascorbic acid in protection against EAE. Free Radic Biol Med 33: 1363–1371. 12419468 10.1016/s0891-5849(02)01048-1

[54] Hayden MR, Tyagi SC (2004) Uric acid: A new look at an old risk marker for cardiovascular disease, metabolic syndrome, and type 2 diabetes mellitus: The urate redox shuttle. Nutr Metab (Lond) 1: 10. 15507132 10.1186/1743-7075-1-10 PMC529248

[55] Spyckerelle Y, Steinmetz J, Deschamps JP (1992) [Comparison of measurements of cholesterol, glucose and uric acid taken at 5-year intervals in children and adolescents]. Arch Fr Pediatr 49: 875–881. 1304153

