# Supplementary figures and images for: Associations of Serum Uric Acid and SLC2A9 Variant with Depressive and Anxiety Disorders: A Population-Based Study
Source: PLoS One. 2013 Oct 29;8(10):e76336. doi: 10.1371/journal.pone.0076336 (PMC3812204; doi:10.1371/journal.pone.0076336)

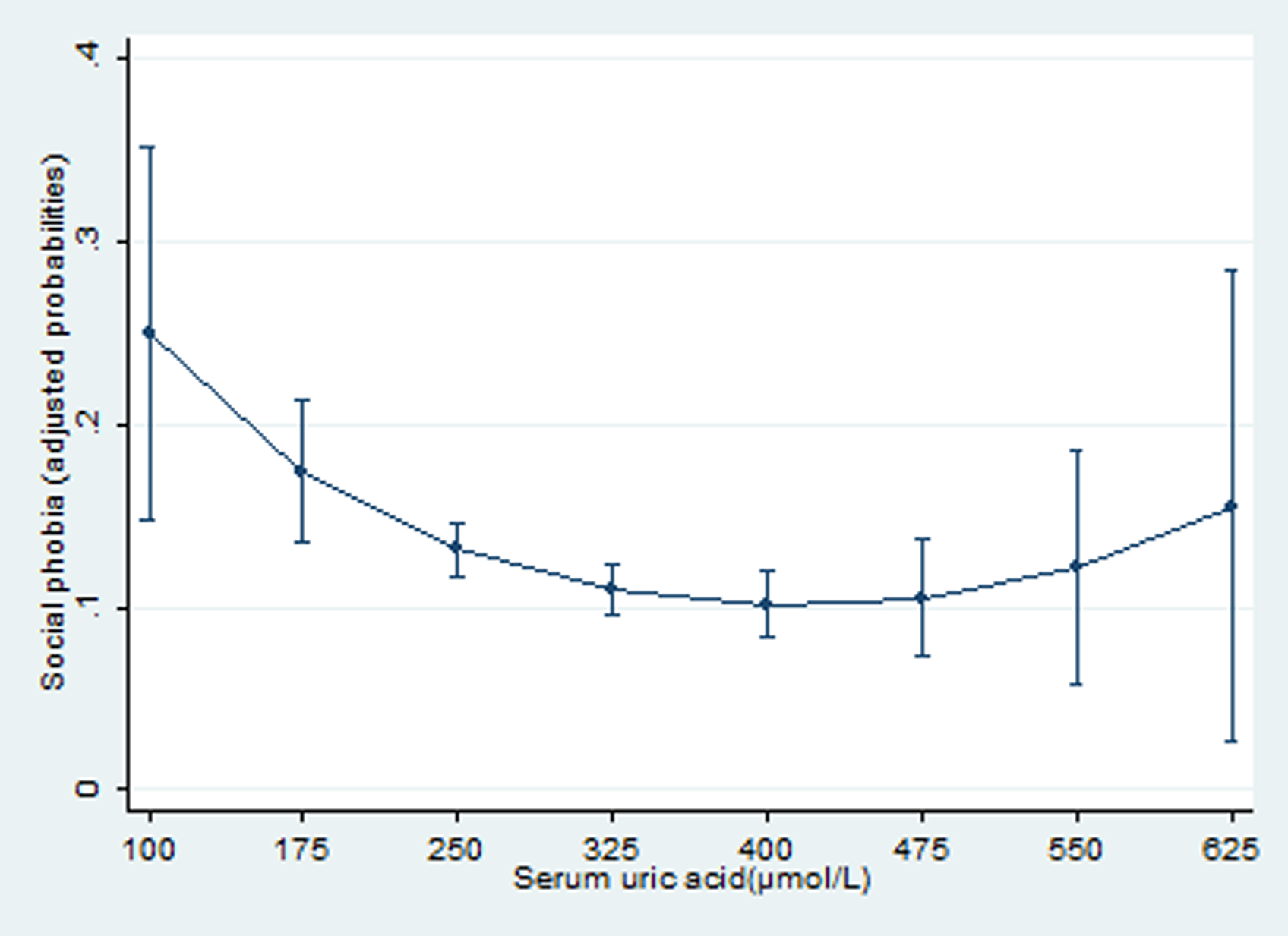

Supplement: Figure S1 — A quadratic curve association between serum uric acid (SUA) and social phobia. The dots and bars represent the adjusted probabilities along with 95%CI of social phobia across SUA levels. Solid line represents the fitted quadratic curve of the effect of SUA on social phobia. Adjusted for age, sex, socio-economic status, alcohol consumption, smoking, diabetes, hypertension, GFR (calculated according to Modification in Diet in Renal Disease equation), drugs that influence uric acid and depression. (TIF) [file pone.0076336.s001.tif]

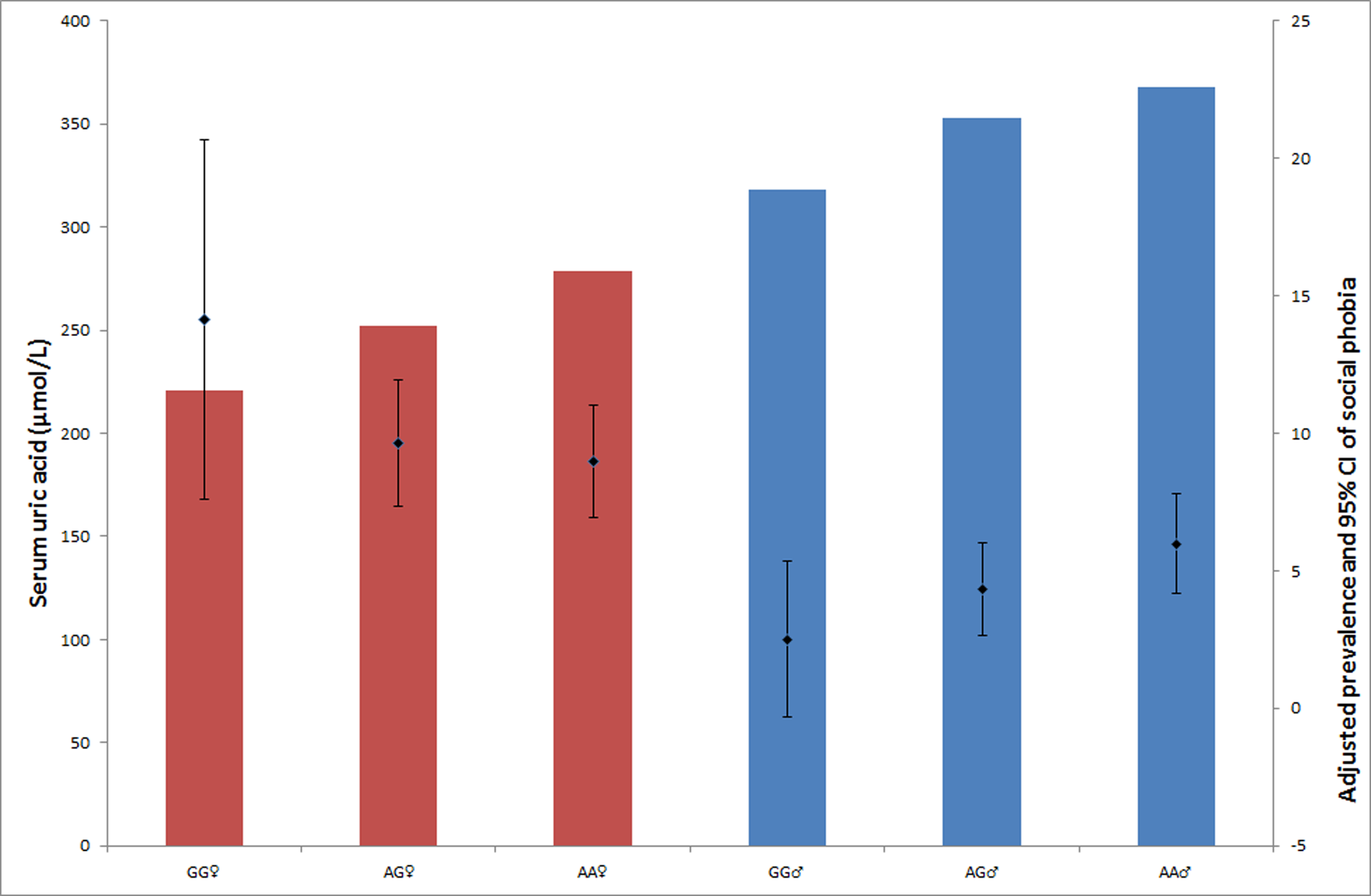

Supplement: Figure S2 — Distribution of serum uric acid (SUA) and current social phobia across genotypes of SLC2A9 rs6855911 in men and women. ♀ = women; ♂ = men. Red and blue barplots indicate mean SUA across the genotypes of SLC2A9 rs6855911 in women and men respectively. Black diamonds with bars indicate adjusted prevalence and 95% CI of social phobia across the genotypes GG, AG and AA of the SLC2A9 rs6855911 variant. Prevalence adjusted for age, sex, socio-economic status, alcohol consumption, smoking, diabetes, hypertension, GFR (calculated according to Modification in Diet in Renal Disease equation), drugs that influence uric acid and depression. (TIF) [file pone.0076336.s002.tif]
