# Supplementary material for: Associations of Serum Uric Acid and SLC2A9 Variant with Depressive and Anxiety Disorders: A Population-Based Study
Source: PLoS One. 2013 Oct 29;8(10):e76336. doi: 10.1371/journal.pone.0076336 (PMC3812204; doi:10.1371/journal.pone.0076336)
Supplement: Table S1 — Distribution of psychiatric disorders across sex-specific quintiles of SUA in males. MDD = major depressive disorder; GAD = generalized anxiety disorder; and SUA = serum uric acid. *P-value<0.05 for quadratic trend tested by a crude logistic model which included a quadratic term (SUA squared) for continuous value of SUA. (DOCX) [file pone.0076336.s003.docx]

**Table S1: Distribution of psychiatric disorders across sex-specific quintiles of SUA in males**

| SUA (mean, µmol/L) | **Q1**  **266** | **Q2**  **318** | **Q3**  **355** | **Q4**  **395** | **Q5**  **472** | **P-trend** |
| --- | --- | --- | --- | --- | --- | --- |
|  | **N(%)** | **N(%)** | **N(%)** | **N(%)** | **N(%)** | **(Test statistic)** |
| **Lifetime pyschiatric disorders** | |  |  |  |  |  |
| *MDD* | *114(34.03)* | *115(33.82)* | *87(25.82)* | *93(31.53)* | *97(32.12)* | *0.420(-0.81)* |
| Mixed MDD | 12(3.58) | 16(4.71) | 12(3.56) | 10(3.39) | 5(1.66) | 0.114(-1.58) |
| Atypical MDD | 9(2.26) | 14(4.12) | 11(3.26) | 11(3.73) | 15(4.97) | 0.219(1.23) |
| Melancholic MDD | 35(10.45) | 31(9.12) | 17(5.04) | 22(7.46) | 29(9.60) | 0.450(-0.75) |
| Unspecified MDD | 58(17.31) | 54(15.88) | 47(13.95) | 50(16.95) | 48(15.89) | 0.761(-0.30) |
| *Any anxiety Disorder* | *62(18.62)* | *39(11.61)* | *39(11.64)* | *29(9.86)* | *40(13.29)* | *0.034(-2.12)** |
| GAD | 6(1.80) | 5(1.49) | 5(1.50) | 3(1.02) | 7(2.33) | 0.808(0.24)* |
| Panic disorder | 7(2.10) | 2(0.60) | 5(1.49) | 10(3.40) | 3(1.00) | 0.805(0.25) |
| Agoraphobia | 8(2.40) | 6(1.79) | 5(1.50) | 6(2.04) | 6(2.01) | 0.810(-0.24) |
| Social phobia | 48(14.41) | 27(8.04) | 29(8.68) | 17(5.78) | 29(9.63) | 0.020(-2.32) |
| **Current psychiatric disorders** | |  |  |  |  |  |
| *MDD* | 60(17.91) | 42(12.35) | 36(10.68) | 46(15.59) | 49(16.23) | 0.923(-0.10) |
| Mixed MDD | 7(2.09) | 7(2.06) | 5(1.48) | 5(1.69) | 5(1.66) | 0.594(-0.53) |
| Atypical MDD | 4(1.19) | 10(2.94) | 6(1.78) | 2(0.68) | 9(2.98) | 0.593(0.53) |
| Melancholic MDD | 23(6.87) | 12(3.53) | 6(1.78) | 11(3.73) | 13(4.30) | 0.137(-1.49) |
| Unspecified MDD | 26(7.76) | 13(3.82) | 19(5.64) | 28(9.49) | 22(7.28) | 0.306(1.02) |
| *Any anxiety Disorder* | 31(9.25) | 22(6.47) | 21(6.23) | 14(4.75) | 24(7.95) | 0.320(-1.00) |
| GAD | 0(0.00) | 0(0.00) | 0(0.00) | 0(0.00) | 0(0.00) | NA |
| Panic disorder | 0(0.00) | 0(0.00) | 0(0.00) | 0(0.00) | 0(0.00) | NA |
| Agoraphobia | 4(1.19) | 6(1.76) | 2(0.59) | 5(1.69) | 3(0.99) | 0.795(-0.26) |
| Social phobia | 27(8.06) | 16(4.71) | 19(5.64) | 10(3.39) | 21(6.95) | 0.379(-0.88) |

MDD= major depressive disorder; GAD=generalized anxiety disorder; and SUA= serum uric acid

*P-value <0.05 for quadratic trend tested by a crude logistic model which included a quadratic term (SUA squared) for continuous value of SUA
