# Supplementary material for: Associations of Serum Uric Acid and SLC2A9 Variant with Depressive and Anxiety Disorders: A Population-Based Study
Source: PLoS One. 2013 Oct 29;8(10):e76336. doi: 10.1371/journal.pone.0076336 (PMC3812204; doi:10.1371/journal.pone.0076336)
Supplement: Table S2 — Distribution of psychiatric disorders across sex-specific quintiles of SUA in females. MDD = major depressive disorder; GAD = generalized anxiety disorder; and SUA = serum uric acid. *P-value<0.05 for quadratic trend tested by a crude logistic model which included a quadratic term (SUA squared) for continuous value of SUA. (DOCX) [file pone.0076336.s004.docx]

**Table S2: Distribution of psychiatric disorders across sex-specific quintiles of SUA in females**

| SUA (mean, µmol/L) | **Q1**  **189** | **Q2**  **232** | **Q3**  **263** | **Q4**  **298** | **Q5**  **370** | **P-trend** |
| --- | --- | --- | --- | --- | --- | --- |
|  | **N(%)** | **N(%)** | **N(%)** | **N(%)** | **N(%)** | **(Test statistic)** |
| **Lifetime psychiatric disorders** | |  |  |  |  |  |
| *MDD* | *234(58.06)* | *208(55.91)* | *209(53.87)* | *166(47.29)* | *154(53.47)* | *0.023(-2.28)* |
| Mixed MDD | 33(8.19) | 28(7.53) | 34(8.76) | 23(6.55) | 30(10.42) | 0.551(0.60) |
| Atypical MDD | 37(9.18) | 31(8.33) | 28(7.22) | 33(9.40) | 25(8.68) | 0.973(-0.03) |
| Melancholic MDD | 69(17.12) | 63(16.94) | 59(15.21) | 42(11.97) | 41(14.24) | 0.066(-1.84) |
| Unspecified MDD | 95(23.57) | 86(23.12) | 88(22.68) | 68(19.37) | 58(20.14) | 0.128(-1.52) |
| *Any anxiety Disorder* | *100(24.88)* | *87(23.64)* | *91(23.51)* | *73(20.92)* | *63(22.03)* | *0.224(-1.21)* |
| GAD | 9(2.24) | 11(2.99) | 9(2.33) | 13(3.72) | 10(3.50) | 0.251(1.15) |
| Panic disorder | 17(4.24) | 15(4.08) | 25(6.48) | 16(4.58) | 11(3.85) | 0.956(0.06) |
| Agoraphobia | 18(4.48) | 20(5.43) | 29(7.49) | 23(6.59) | 14(4.90) | 0.485(0.70) |
| Social phobia | 78(19.40) | 57(15.49) | 55(14.21) | 38(10.89) | 41(14.34) | 0.008(-2.64) |
| **Current psychiatric disorders** | |  |  |  |  |  |
| *MDD* | 103(25.56) | 84(22.58) | 94(24.23) | 71(20.23) | 72(25.00) | 0.523(-0.64) |
| Mixed MDD | 17(4.22) | 14(3.76) | 18(4.64) | 9(2.56) | 19(6.60) | 0.413(0.82) |
| Atypical MDD | 16(3.97) | 12(3.23) | 10(2.58) | 16(4.56) | 12(4.17) | 0.638(0.47) |
| Melancholic MDD | 32(7.94) | 31(8.33) | 32(8.25) | 23(6.55) | 14(4.86) | 0.094(-1.67) |
| Unspecified MDD | 38(9.43) | 27(7.26) | 34(8.76) | 23(6.55) | 27(9.39) | 0.746(-0.32) |
| *Any anxiety Disorder* | 66(16.38) | 49(13.17) | 57(14.69) | 36(10.26) | 30(10.42) | 0.009(-2.61) |
| GAD | 0(0.00) | 0(0.00) | 0(0.00) | 0(0.00) | 0(0.00) | NA |
| Panic disorder | 0(0.00) | 1(0.27) | 0(0.00) | 0(0.00) | 0(0.00) | NA |
| Agoraphobia | 12(2.98) | 11(2.96) | 21(5.41) | 13(3.70) | 7(2.43) | 0.933(0.08) |
| Social phobia | 58(14.39) | 39(10.48) | 40(10.31) | 24(6.84) | 24(8.33) | 0.001(-3.21) |

MDD= major depressive disorder; GAD=generalized anxiety disorder; and SUA= serum uric acid

*P-value <0.05 for quadratic trend tested by a crude logistic model which included a quadratic term (SUA squared) for continuous value of SUA
