# Supplementary material for: Associations of Serum Uric Acid and SLC2A9 Variant with Depressive and Anxiety Disorders: A Population-Based Study
Source: PLoS One. 2013 Oct 29;8(10):e76336. doi: 10.1371/journal.pone.0076336 (PMC3812204; doi:10.1371/journal.pone.0076336)
Supplement: Table S3 — Crude and adjusted logistic regression analysis of SUA (per 100 µmol/L) and psychiatric disorders in males. MDD = major depressive disorder; GAD = generalized anxiety disorder; and SUA = serum uric acid.If P value for the quadratic term (SUA2) is significant, ORs derived from models with quadratic term are presented.Adjusted for age, sex, socio-economic status, alcohol consumption, smoking, diabetes, hypertension, GFR (calculated according to Modification in Diet in Renal Disease equation) and drugs that influence uric acid and additionally for anxiety (in the association between SUA and depression) and depression (in the association between SUA and anxiety). *Logistic regression not possible due to zero prevalence; NA = not available. (DOCX) [file pone.0076336.s005.docx]

**Table S3: Crude and adjusted logistic regression analysis of SUA (per 100µmol/L) and psychiatric disorders in males**

|  | **Crude OR(95% CI)** | | | | **Adjusted OR(95% CI)** | | | |
| --- | --- | --- | --- | --- | --- | --- | --- | --- |
| **Psychiatric disorders** | SUA | Pvalue_SUA_ | SUA^2^ | Pvalue_SUA2_ | SUA | Pvalue_SUA_ | SUA^2^ | Pvalue_SUA2_ |
| **Lifetime psychiatric disorders** | |  |  |  |  |  |  |  |
| *MDD* | *1.00(0.87-1.15)* | *0.995* | *-* | *-* | *1.01(0.87-1.19)* | *0.865* | *-* | *-* |
| Mixed MDD | 0.82(0.56-1.20) | 0.310 | - | - | 0.79(0.52-1.18) | 0.241 | - | - |
| Atypical MDD | 1.23(0.88-1.73) | 0.226 | - | - | 1.31(0.91-1.87) | 0.143 | - | - |
| Melancholic MDD | 0.97(0.76-1.24) | 0.826 | - | - | 0.94(0.72-1.21) | 0.615 | - | - |
| Unspecified MDD | 1.00(0.84-1.20) | 0.959 | - | - | 1.03(0.85-1.26) | 0.74 | - | - |
| *Any anxiety disorder* | *0.72(0.54-0.95)* | *0.021* | *1.18(1.01-1.39)* | *0.042* | *0.71(0.53-0.96)* | *0.025* | *1.17(1.00-1.38)* | *0.056* |
| GAD | 0.71(0.37-1.39) | 0.32 | 1.42(1.04-1.93) | 0.026 | 0.67(0.32-1.39) | 0.283 | 1.37(0.99-1.88) | 0.055 |
| Panic disorder | 1.02(0.61-1.70) | 0.948 | - | - | 0.95(0.54-1.69) | 0.870 | - | - |
| Agoraphobia | 0.98(0.61-1.59) | 0.943 | - | - | 0.84(0.50-1.39) | 0.492 | - | - |
| Social phobia | 0.84(0.67-1.07) | 0.154 | - | - | 0.86(0.67-1.11) | 0.241 | - | - |
| **Current psychiatric disorders** | |  |  |  |  |  |  |  |
| *MDD* | *0.84(0.64-1.11)* | *0.233* | *1.17(1.00-1.36)* | *0.05* | *0.83(0.62-1.12)* | 0.234 | 1.16(0.99-1.35) | 0.067 |
| Mixed MDD | 1.01(0.61-1.66) | 0.973 | - | - | 0.94(0.56-1.57) | 0.808 | - | - |
| Atypical MDD | 1.27(0.80-2.02) | 0.307 | - | - | 1.32(0.80-2.16) | 0.275 | - | - |
| Melancholic MDD | 0.86(0.61-1.21) | 0.377 | - | - | 0.85(0.58-1.23) | 0.378 | - | - |
| Unspecified MDD | 1.11(0.86-1.44) | 0.423 | - | - | 1.09(0.82-1.45) | 0.539 | - | - |
| *Any anxiety disorder* | *0.96(0.74-1.25)* | *0.765* | *-* | *-* | *0.97(0.73-1.29)* | *0.839* | - | - |
| GAD* | NA | NA | NA | NA | NA | NA | NA | NA |
| Panic disorder* | NA | NA | NA | NA | NA | NA | NA | NA |
| Agoraphobia | 0.90(0.49-1.66) | 0.735 | - | - | 0.81(0.43-1.52) | 0.506 | - | - |
| Social phobia | 0.98(0.74-1.31) | 0.918 | - | - | 1.02(0.75-1.38) | 0.918 | - | - |

MDD= major depressive disorder; GAD=generalized anxiety disorder; and SUA= serum uric acid

If P value for the quadratic term (SUA^2^) is significant, ORs derived from models with quadratic term are presented.

Adjusted for age, sex, socio-economic status, alcohol consumption, smoking, diabetes, hypertension, GFR (calculated according to Modification in Diet in Renal Disease equation) and drugs that influence uric acid and additionally for anxiety (in the association between SUA and depression) and depression (in the association between SUA and anxiety).

*Logistic regression not possible due to zero prevalence; NA=not available
